# Parental leadership in motion: neural and behavioral mechanisms of synchrony during parent–child interaction

**DOI:** 10.1093/scan/nsag066

**Published:** 2026-08-08

**Authors:** Omer Katz, Andrey Markus, Simone Shamay-Tsoory

**Affiliations:** School of Psychology, University of Haifa, Abba Hushi Ave 199, Haifa, 3498838, Israel; School of Psychology, University of Haifa, Abba Hushi Ave 199, Haifa, 3498838, Israel; The Integrated Brain and Behavior Research Center (IBBRC), University of Haifa, Abba Hushi Ave 199, Haifa, 3498838, Israel; School of Psychology, University of Haifa, Abba Hushi Ave 199, Haifa, 3498838, Israel; The Integrated Brain and Behavior Research Center (IBBRC), University of Haifa, Abba Hushi Ave 199, Haifa, 3498838, Israel

**Keywords:** parent–child interaction, interbrain synchrony, hyperscanning, behavioral synchrony

## Abstract

Parent–child synchrony, the coordination of movements and goals between parents and children, plays a vital role in social development, yet its neural mechanisms and leadership dynamics remain unclear. Using functional near-infrared spectroscopy hyperscanning, 33 parent–child dyads (children 8–15 years) played a tablet-based game (“The Flocking Game”) under spontaneous and intentional synchrony instructions. Results revealed that dyads exhibited behavioral synchrony values significantly greater than zero (with zero reflecting no synchrony) even without instruction, and synchrony increased when coordination of movement was explicitly required. Parents led more during spontaneous play, reflecting their natural regulatory role, whereas leadership equalized under intentional instructions. Neural analyses revealed significant inter-brain synchrony across the dorsolateral prefrontal cortex (DLPFC), inferior frontal gyrus (IFG), and inferior parietal lobule (IPL). In the intentional condition, stronger parent left-DLPFC coupling with the child IFG or IPL predicted greater behavioral synchrony, while spontaneous synchrony was linked to parent IPL-child IFG synchrony. These results suggest that spontaneous synchrony relies on mirroring within the action observation network, whereas intentional synchrony engages executive control, highlighting complementary neural pathways through which parental leadership shapes goal-directed interaction.

## Introduction

Parent–child interactions play a central role in children’s social, emotional, and cognitive development (Bowlby 1969, Harrist and Waugh 2002). Consistent with this view, the quality of the parent–child relationship predicts key developmental outcomes, including social competence and emotion regulation (Black and Logan 1995, Brumariu 2015). Although relationship quality is not determined solely by interactional behavior, it becomes observable through repeated parent–child interactions (Biringen 2000).

Parent–child relationships are inherently asymmetrical, with parents fulfilling roles of authority, guidance, and regulation, and children occupying positions of dependence and responsiveness (Popper and Mayseless 2003). This asymmetry evolves across development, as children gain autonomy while parents continue to serve regulatory and guiding roles, particularly in structured, uncertain, or emotionally demanding contexts (Jones *et al.* 2015, Bosmans *et al.* 2022). These dependencies persist in changing form throughout development, and children tend to follow parental guidance, seeking stability and support from early childhood through adolescence (Popper and Mayseless 2003, Jones *et al.* 2015). When a sense of security is provided by the leader, it facilitates the fulfillment of existing regulatory and affiliative needs and enables the activation of other behavioral systems, such as exploration (Bowlby 1977), or supports engagement with higher-order needs (Burns 2012). Such regulatory dynamics are reflected not only in overt communication and decision-making but also in more subtle interpersonal processes, such as synchrony (Feldman 2007).

Synchrony refers to the coordination of actions, movements, emotions, goals, and physiological processes between individuals and constitutes a pervasive feature of social interaction (Bernieri and Rosenthal 1991, Ackerman and Bargh 2010, Palumbo *et al.* 2017). Such coordination may arise intentionally, as in task-driven collaboration such as rowing or dancing, or spontaneously, as in rhythmic clapping (Schmidt *et al.* 1998, Repp and Penel 2004, Schmidt and Richardson 2008). From an evolutionary and developmental perspective, interpersonal synchrony functions as an adaptive mechanism that enhances social bonding, mutual understanding, and group cohesion, often described as social glue (Chartrand and Lakin 2013, Tarr *et al.* 2016). It promotes affiliation and likability (Hove and Risen 2009), whereas psychological distancing attenuates these effects by disrupting synchrony (Miles *et al.* 2010).

One way to examine behavioral synchrony within asymmetrical parent–child dyads is by assessing leader-follower dynamics during joint tasks. Yarmolovsky and Geva (2024) examined parent–child motor synchrony during cooperative and competitive play using a turn-based tower-building (Jenga©) task. In the cooperative condition, dyads worked together to maintain tower stability, with success defined at the dyadic level. In the competitive condition, participants played against each other under the same turn-taking rules, but responsibility for collapse was individualized. The authors observed comparable leadership in cooperative play but a clear asymmetry in competition: parents showed greater parent-led in-phase synchrony, whereas children exhibited more anti-phase synchrony when leading. This suggests that competitive contexts amplify parental leadership, consistent with attachment-based models proposing that leadership is context-sensitive and more pronounced under stress or uncertainty (Mayseless and Popper 2019).

To characterize the neural mechanisms underlying synchrony in parent–child dyads, it is necessary to consider the brain systems that have been proposed to support interpersonal coordination. The first system found to support synchrony is the action observation network, encompassing the inferior frontal gyrus (IFG) and inferior parietal lobule (IPL), which have been proposed to form part of a mirror neuron system hypothesized to support the mapping of observed actions onto one’s own motor representations (Buccino *et al.* 2004). The IFG has been suggested to play a role in mapping others’ actions onto one’s own motor representations (Rizzolatti and Craighero 2004), and interbrain synchrony in the IFG has been reported during social alignment tasks such as dialogue (Jiang *et al.* 2012). The IPL has likewise been proposed as a functionally integrated node within this network, supporting the mapping of observed actions onto the motor system and contributing to shared neural representations (Gatti *et al.* 2017, Hardwick *et al.* 2018). Neuroimaging studies of social touch showed overlapping IPL activation between interacting partners, particularly among empathizers holding a partner’s hand in pain (Korisky *et al.* 2020).

The second system implicated in interpersonal synchrony is the fronto-parietal network, anchored in the dorsolateral prefrontal cortex (DLPFC), which has been proposed to support deliberate monitoring and regulation of joint behavior. As a central component of executive control and emotion-regulation systems, the DLPFC exerts top-down modulation over limbic and sensorimotor circuits to maintain goal-directed control (Wager and Smith 2003, Barbey *et al.* 2013) and has been associated with cognitive reappraisal, stress regulation, and attenuation of distress during emotionally charged interactions (Goldin *et al.* 2008). In interpersonal contexts, DLPFC interbrain synchrony predicts successful co-regulation of distress, suggesting shared activation may support dyadic regulatory coordination (Avnor *et al.* 2025), and at the group level has been associated with reduced extremism during deliberation (Sobeh and Shamay-Tsoory 2025), highlighting its role in reflective, controlled engagement.

**Figure 1:**
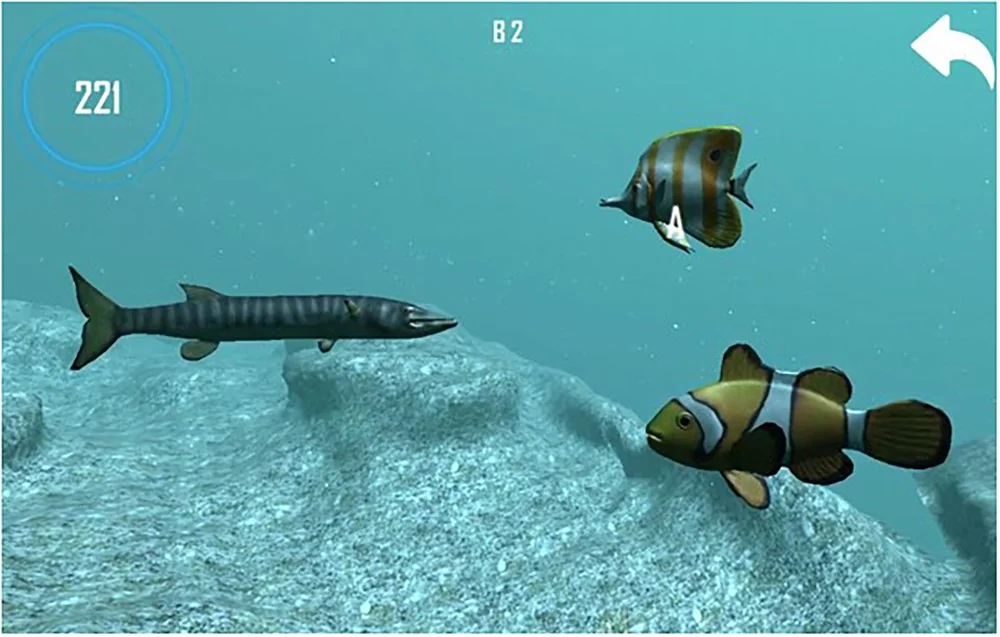
The Flocking Game, as played by a dyad of two participants. Both participants are positioned side-by-side, oriented toward the left, and navigate in front of a moving obstacle. A live score is continuously displayed in the upper-left corner of the screen.

The purpose of the present study was to investigate the behavioral and neural dynamics of parent–child synchrony by comparing spontaneous synchrony, emerging without explicit instruction, with intentional synchrony guided by task demands. We examined synchrony within a gaming context that provides a naturalistic setting for real-time coordination through shared goals, turn-taking, and mutual responsiveness (Bergen 2002), and assessed how leadership manifests across conditions and is reflected at the neural level. To that end, we combined hyperscanning functional near-infrared spectroscopy (fNIRS) with a novel interactive game requiring navigation of obstacles and collection of bonus objects, compelling dyads to balance synchrony with goal-directed action (Fig. 1).

First, to validate the task, we measured behavioral indices (game score, obstacle collisions, goal collection, and Euclidean distance) across conditions. Following validation, our first hypothesis was that dyads would exhibit meaningful behavioral interpersonal synchrony, with synchrony levels significantly exceeding zero. We further anticipated greater synchrony in the intentional condition, where participants were explicitly instructed to coordinate, compared to the spontaneous condition, which relied on natural task-driven collaboration. Consistent with the asymmetrical structure of the parent–child relationship, our second hypothesis was that parents would demonstrate greater leadership than children in the spontaneous condition, where task demands are less explicit and require more autonomous regulation. In contrast, we expected no significant leadership difference in the intentional condition, as clearer instructions may reduce the need for parental guidance.

At the neural level, our third hypothesis was that interbrain synchrony would be more pronounced in the intentional condition than in the spontaneous condition and that behavioral synchrony would be associated with stronger interbrain synchrony within the action-observation network and the DLPFC.

## Materials and methods

### Participants

An a priori power analysis using G*Power 3.1.9.6 (Faul *et al.* 2007) determined the required sample size. The main analysis examined whether a significant behavioral synchrony (all sync) in both conditions (spontaneous and intentional) had appeared, by comparing both condition’s value to zero, for parents and children separately. For one-sample *t*-tests comparing each condition to zero, with an expected medium effect size of Cohen’s *d* = 0.5, α  =  0.05, and desired power of 0.80, the required sample size was *N* = 27 pairs (54 individuals). A post hoc power analysis indicated that the final sample (*N* = 33 dyads) achieved sufficient power (≥80%) to detect medium-sized within-subject effects.

A total of 37 parent–child dyads (*N* = 74 individuals) were initially recruited for the study. Four dyads were excluded from analysis due to recording difficulties, resulting in a final sample of 33 dyads. Within the retained dataset, 1.06% of the WTC data were excluded during preprocessing, either because they exceeded ±3.5 standard deviations from the mean of the subject, in which case specific ROI combinations in specific blocks were removed, or due to excessive channel rejection caused by poor signal quality, in which case entire participants’ recordings were removed. Child participants were aged 8 to 15 years (20 females), and parents ranged from 38 to 52 years (26 females), with no age restrictions imposed. Dyads were recruited via targeted Facebook advertisements and received monetary compensation. All participants were native Hebrew speakers without visual impairments and reported no history of neurological or psychiatric conditions, learning disabilities, or attention difficulties. Written informed consent was obtained in accordance with the Institutional Review Board of the University of Haifa (approval no. 042/23).

### Experimental task and conditions

The experimental paradigm employed a novel tablet-based application developed in our lab, termed “The Flocking Game,” designed to assess real-time coordination and movement synchrony between two participants. Each player selected a unique fish avatar and controlled it via touchscreen, allowing free movement along the horizontal and vertical axes. At the start of each 60-second session, the two fish appeared side by side, facing left, and could navigate freely within the underwater scene. Shortly thereafter, moving obstacles depicted as fish swimming toward the right emerged from the left side of the screen, requiring players to avoid collisions. Stars appeared occasionally as bonus items that could be collected to increase score.

The scoring system was designed to capture cooperative dynamics, with each dyad’s total score determined by three components:

1. Obstacle avoidance—every collision with an obstacle resulted in point deductions.
2. Bonus collection—colliding with bonus items increased the shared score.
3. Movement synchrony—the degree of real-time alignment in dyadic motion further contributed to overall performance.

Participants played the game under two experimental conditions:

#### Spontaneous synchrony condition

In the initial phase, participants were instructed to avoid obstacles and collect bonus items, without being informed that movement synchrony affected scoring. They completed four 60-second rounds with increasing difficulty, manipulated by varying the number, speed, and angle of incoming obstacles.

#### Intentional synchrony condition

After these rounds, participants were informed of an additional rule: beyond avoiding obstacles and collecting bonuses, they were required to move in synchrony. Bonus items were collected only if both players touched them simultaneously. The same four difficulty levels were then repeated under this instruction set.

Throughout the experiment, dyads sat side-by-side, each holding their own tablet. Although physically proximate, they were instructed not to communicate with each other during gameplay or between rounds.

### Procedure

After selecting the appropriate cap size based on individual head measurements, fNIRS caps were fitted, followed by a signal optimization phase to ensure high-quality neural data collection. Once the setup was completed, participants were asked to sit quietly for a 60-second baseline block, during which they were instructed not to speak or move. Interbrain synchrony was measured during this period and served as a baseline for subsequent analyses. After the baseline block, participants performed the spontaneous condition and then the intentional condition. The entire session, including setup, instructions, and gameplay, lasted approximately 45 minutes.

### Behavioral measures

Game data were automatically logged by the server, including each player’s raw avatar position in real time at approximately 50 frames per second (FPS), as well as the positions of goals and obstacles. From these raw data, the server calculated the game scores presented at play-time based on motion synchrony parameters (positive addition), goal collection (bonus points), and obstacle collisions (penalties), and the position data were stored in the server and retrieved for post-hoc offline analyses.

From the stored data, we calculated several performance measures per game: mean Euclidian distance between avatars over time, number of goals collected, number of obstacle collisions, final game score, and motion synchrony.

Motion synchrony in the Flocking Game was quantified using the cosine of velocity vectors (CVV), a time-resolved measure of interpersonal synchrony developed for high-resolution motion tracking data (Reiss *et al.* 2019). The instantaneous velocity of each avatar was computed by differentiating its positional trajectory over time, yielding a direction-specific estimate at each time point. Synchrony was defined as the cosine of the angle between the two velocity vectors, with values near 1 indicating aligned directions, values near 0 independent movement, and negative values opposing directions.

Although the game presents a three-dimensional visual environment, avatar movement was constrained to the horizontal plane; accordingly, the depth (z-axis) was held constant and CVV was calculated from x- and y-axis movement only, consistent with Reiss *et al.* (2019). To reduce noise and capture sustained coordination, CVV values were aggregated across time within each round.

Leader–follower dynamics were assessed by incorporating a temporal lag of up to 1000 ms between velocity vectors, systematically shifting one player’s time series relative to the other. This procedure identified time points at which one participant’s movement preceded the other’s and enabled calculation of the proportion of time the parent led the child, and vice versa, within each round. These leadership percentages served as behavioral indices of dyadic coordination in subsequent analyses.

### Neural data acquisition

Neural data were acquired using the NIRx fNIRS system to simultaneously measure cortical oxygenated hemoglobin (O_2_Hb) and deoxygenated hemoglobin (HHb) concentrations in dyads. The system employed 16 light sources and 23 detectors, yielding 46 channels, including 38 long-separation channels (30 to 45 mm source-detector distance) and eight short-separation channels with limited penetration depth. Long-separation channels detected hemoglobin concentration changes up to approximately 15 to 20 mm below the scalp, capturing outer cortical regions while also reflecting superficial tissue signals (Toga and Thompson 2003, Brigadoi and Cooper 2015). In contrast, short-separation channels primarily detected extracerebral activity, including blood pressure variations (e.g. Mayer waves), respiration, and cardiac cycles, which affect both cortical and superficial blood flow. Signals from these short channels were used to regress out superficial physiological noise, thereby enhancing isolation of cortical activity (Pinti *et al.* 2020).

**Figure 2:**
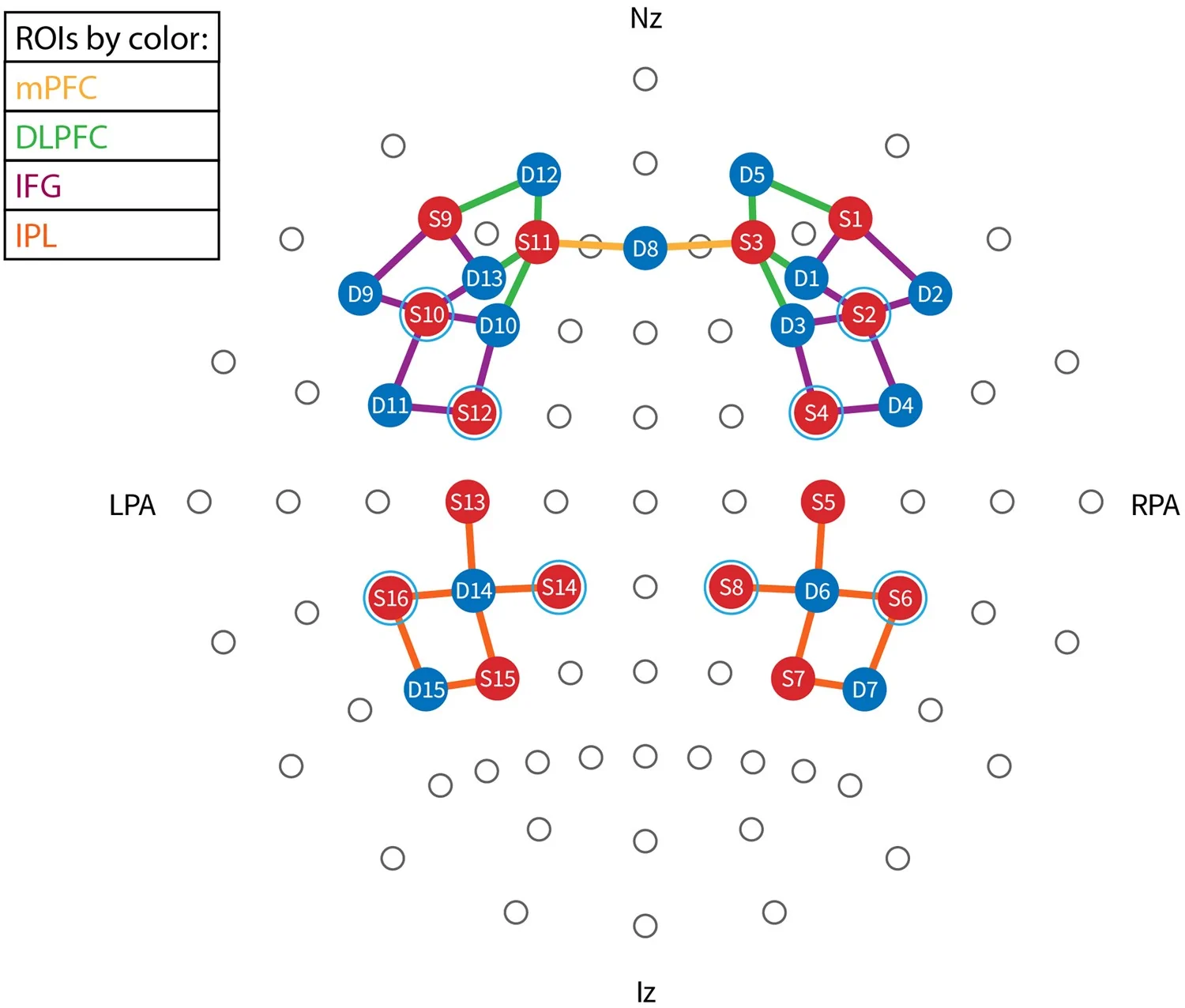
Optode arrangement on the fNIRS caps, presented as a 2D layout generated in the NirSite application (NIRx), where red circles represent sources, blue circles represent detectors, and the connecting lines between them indicate the channels. Blue circles encircling certain sources represent short-separation channels. Channel colors correspond to the specific regions of interest to which each channel was assigned.

**Figure 3:**
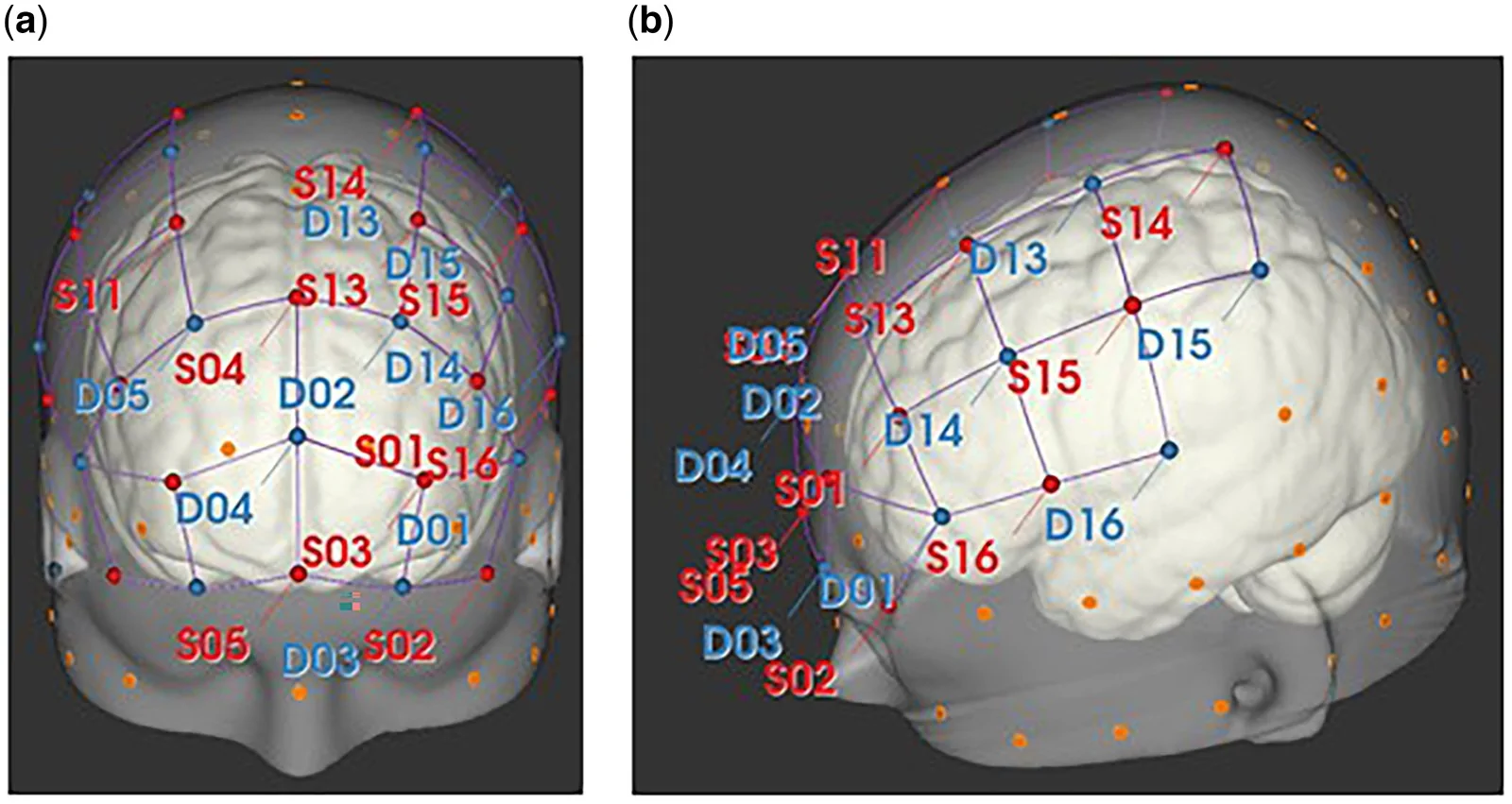
Illustration of the optode arrangement visualized in 3D using the Aurora software (NIRx), shown in frontal (a) and lateral (b) views. Red circles labeled with “S” indicate sources, and blue circles labeled with “D” represent detectors. The numbers adjacent to each label denote the corresponding source or detector number.

Channels were defined by optode placement over the IFG, IPL, medial prefrontal cortex (mPFC), and DLPFC, guided by the standard 10-20 system anchor points, with coordinates defined using NIRx’s Aurora Program. Optode placement was further informed by the fNIRS Optodes’ Location Decider (fOLD) (Zimeo Morais *et al.* 2018), which provides a systematic mapping between fNIRS channel locations and underlying cortical regions based on Brodmann-area-based ROIs and corresponding Montreal Neurological Institute (MNI) coordinates (Table 1 and Figs 2 and 3). Signals from multiple channels within each ROI were averaged to yield a single regional time series for subsequent interbrain synchrony and brain–behavior analyses.

Participants were fitted with caps appropriate to head diameter (52 to 58 cm). Cortical hemodynamic responses were recorded using continuous-wave fNIRS at wavelengths of 760 and 850 nm, corresponding to HHb and O_2_Hb sensitivity, respectively. O_2_Hb was sampled at approximately 5 Hz using the Aurora software.

### Data preprocessing

Data pre-processing was done using Satori software (Brain Innovation, Maastricht, NL). The process began with the conversion of raw intensity data into optical density values. Motion artifacts were corrected using temporal derivative distribution repair (TDDR), a robust motion-correction method that identifies and attenuates motion-related artifacts based on outliers in the temporal derivative of the signal (Fishburn *et al.* 2019). In a separate step, spike artifacts were identified as values exceeding ±3.5 standard deviations from the mean signal and were removed accordingly. A bandpass filter (0.01–0.2 Hz) was applied to remove physiological noise, such as heart rate and respiration, while retaining the hemodynamic signals. Optical density data were subsequently converted into O_2_Hb and HHb concentrations using the modified Beer-Lambert law to extract hemodynamic signals. Both O_2_Hb and HHb signals were extracted and examined; O_2_Hb was selected as the primary signal for interbrain synchrony analyses, while parallel analyses were conducted on HHb signals to assess consistency across hemoglobin measures. channel-level data quality control was performed using the Scalp Coupling Index (SCI)-based preprocessing filter implemented in Satori (Brain Innovation, Maastricht, NL), which was selected a priori to identify channels with poor optode-scalp coupling or low signal quality. Channels with an SCI value ≤ 0.75 were excluded from subsequent analyses (Pollonini *et al.* 2016). Across participants, the average number of rejected channels was six, with a minimum of zero and a maximum of 15 rejected channels per participant. In addition, Satori applies a hemodynamic plausibility filter that evaluates the correlation between O_2_Hb and HHb signals, and channels exhibiting abnormally high positive O_2_Hb–HHb correlations were excluded (Cui *et al.* 2010). For each channel, short separation regression was carried out using its nearest short separation counterpart.

### Wavelet transform coherence

We used WTC to assess inter- and intra-brain synchrony. Analyses focused on frequencies between 0.03 Hz (33 s period) and 0.16 Hz (6 s period) to capture the hemodynamic response while excluding respiratory and cardiac noise. Coherence was computed for each suggested ROI combination between participants’ IFG, IPL, mPFC, and DLPFC, as well as for all possible ROI combinations within individual brains, using MATLAB’s Wavelet Coherence Toolbox (Grinsted *et al.* 2004).

Because the original fNIRS time series were continuous across each session, WTC vectors were segmented into time windows corresponding to the nine significant epochs: one baseline block, four spontaneous synchrony blocks, and four intentional synchrony blocks. WTC values were averaged within each block to yield a single mean coherence value per block for each ROI combination. For each dyad × ROI combination, the mean baseline WTC value was subtracted from each corresponding game block, reducing random inter-dyad variance. These baseline-corrected values were used in subsequent analyses and selected ΔWTC.

### Statistical analysis

#### Behavioral data analyses

Behavioral synchrony data were analyzed in R (version 4.5.1; Baayen *et al.* 2008). To assess task performance, mean Euclidean distance, goal collection, obstacle collisions, and game score were calculated for each dyad across blocks within each condition (spontaneous, intentional) and compared using paired-sample *t*-tests.

For behavioral synchrony, an overall synchrony index was computed per condition and tested against zero using single-sample *t-*tests, then compared between conditions with paired-sample *t*-tests. To examine synchrony components, data were separated into perfect synchrony (lag = 0) and lagged synchrony, averaged within condition, and compared using paired-sample *t*-tests.

Leadership differences were analyzed using linear mixed effects (LME) models implemented in the lme4 package (Bates *et al.* 2007), with condition (intentional, spontaneous) and family role (parent, child) as fixed factors, block number and dyad number as random factors, and lead ratio as the dependent variable. All comparisons were false discovery rate (FDR) corrected (Benjamini and Hochberg 1995).

#### Neural data analyses

To identify the neural underpinnings of interbrain synchrony, we first tested which ΔWTC values (task-related WTC values minus baseline) were significantly greater than zero across ROI combinations and conditions. A LME model was constructed with condition (spontaneous, intentional) and ROI combination (49 in total) as fixed factors, dyad number as a random factor, and ΔWTC as the dependent variable. Post hoc contrasts within the model were performed to determine which ROI combinations showed significantly above-baseline synchrony, and Bonferroni correction was applied for multiple comparisons.

In the second analytic step, we examined the relationship between neural synchrony and behavioral synchrony using stepwise multiple regression analyses. ROI combinations that showed significantly above-baseline ΔWTC in the previous analysis were entered as predictors, and the behavioral synchrony (CVV All Sync) measure served as the dependent variable. Separate stepwise regression models were conducted for the intentional and spontaneous conditions to identify which ROI combinations uniquely predicted behavioral synchrony. All comparisons were Bonferroni-corrected for multiple comparisons.

## Results

**Figure 4:**
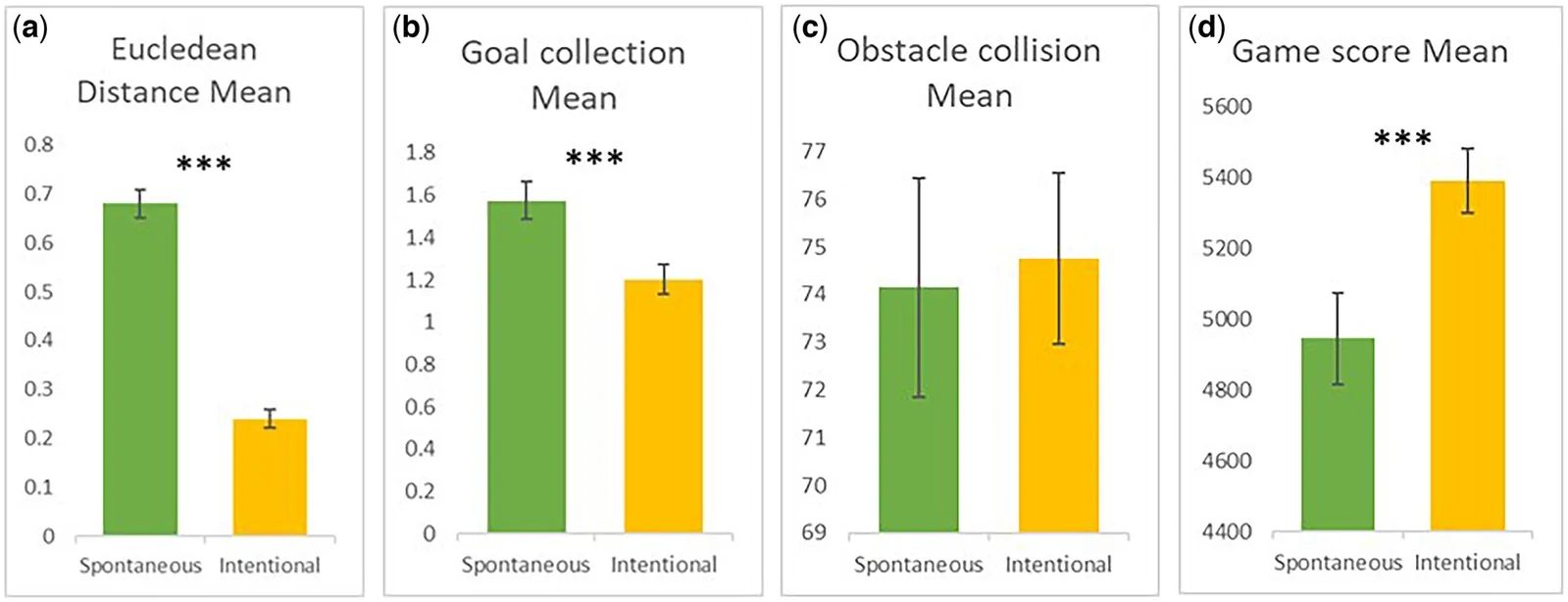
Comparison of behavioral measure values between the spontaneous and the international conditions: Euclidean distance mean (a), goal collection mean (b), obstacle collision mean (c), game score mean (d). ***Indicates significance at *P* < .001.

We averaged each behavioral parameter value across the blocks of each condition and then conducted a series of paired-sample t-tests within the groups between the intentional and spontaneous conditions, on each of the behavioral measures (Fig. 4). First, we found that Euclidean distance in the intentional condition (*M* = .24, SD = .114) was significantly smaller (*t*_(36)_ = 16.50, *P* < .001) than that in the spontaneous condition (*M* = .68, SD = .17). For the goal collection measure, we found a significant difference between intentional and spontaneous conditions (*t*_(36)_ = 3.88, *P* < .001) such that the number of goals collected in the spontaneous condition (*M* = 1.574, SD = .529) was significantly larger than that in the intentional condition (*M* = 1.202, SD = .432). In contrast, the number of obstacle collisions did not differ significantly between conditions (*t*_(36)_ = −.40, *P* = .687), such that the number of obstacle collisions in the intentional condition (*M* = 74.75, SD = 10.796) was not significantly larger than that in the spontaneous condition (*M* = 74.148, SD = 13.831). Lastly, we found that game score in the intentional condition (*M* = 5388.318, SD = 539.612) was significantly higher (*t*_(36)_ = 4.15, *P* < .001) than that in the spontaneous condition (*M* = 4944.628, SD = 769.549).

To test the overall synchrony between the two conditions, a paired-sample *t*-tests between conditions were used, which found a significant difference between intentional and spontaneous conditions (*t*_(33)_ = −10.28, *P* < .001) such that overall synchrony in the intentional condition (*M* = .749, SD = .097) was significantly greater than in the spontaneous condition (*M* = .543, SD = .972).

These findings suggest that, when explicitly instructed to synchronize, dyads shifted their focus toward synchrony, sometimes at the expense of task efficiency (.

To test our first hypothesis regarding the behavioral synchrony levels, a single-sample *t*-test was used for each condition to compare its overall synchrony to zero. This examination found that in the spontaneous condition (*M* = .537, SD = .14), synchrony was significantly above zero (*t*_(144)_ = 46.08, *P* < .001), while in the intentional condition (*M* = .738, SD = .131) synchrony was also significantly greater than zero, *t*_(147)_ = 68.17, *P* < .001. These results confirm that spontaneous synchrony reliably emerged even without explicit instructions and that intentional coordination further amplified the degree of synchrony, as expected.

**Figure 5:**
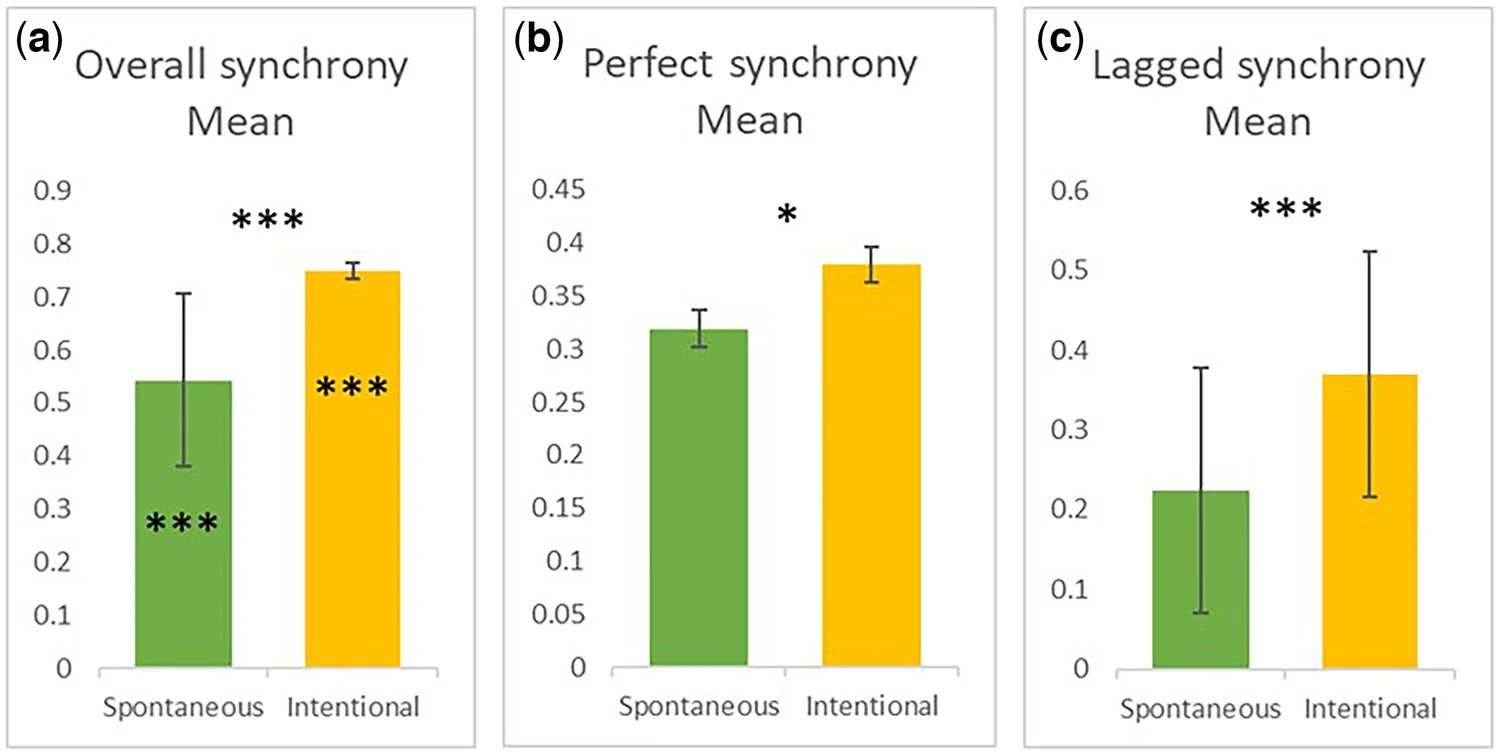
Synchrony components value differences between the spontaneous and the international conditions: Overall synchrony mean (a), perfect synchrony mean (b), lagged synchrony mean (c). *Indicates significance at *P* < .05 while *** indicates significance at *P* < .001.

To test our second hypothesis, regarding perfect (lag = 0) and lagged synchrony components, we averaged each of the synchrony components across the blocks of each condition and then conducted a series of paired-sample *t*-tests within the groups between the intentional and spontaneous conditions (Fig. 5).

For the perfect synchrony component, we found a significant difference between intentional and spontaneous conditions (*t*_(33)_ = −2.45, *P* = .002) such that perfect synchrony in the intentional condition (*M* = .379, SD = .100) was significantly greater than in the spontaneous condition (*M* = .319, SD = .105).

Likewise, in the lagged synchrony component, a significant difference was found (*t*_(33)_ = −8.27, *P* < .001), such that the component in the intentional condition (*M* = .37, SD = .92) was significantly larger (*t*_(36)_ = 16.50, *P* < .001) than that in the spontaneous condition (*M* = .224, SD = .92).

**Figure 6:**
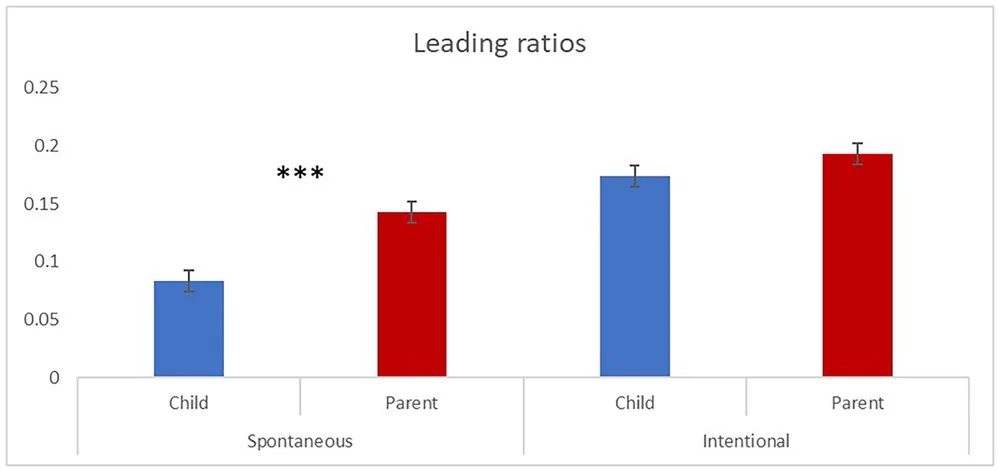
Lead ratio differences between parents and children in the spontaneous and the international conditions. ***Indicates significance at *P* < .001.

To determine whether one member of the dyad (parent or child) led more than the other, in each condition, we used LME models using the R language, and the lme4 package for the R language (Bates *et al.* 2007). We constructed a model in which condition (intentional, spontaneous) and family role (parent, child) were fixed factors; block number and dyad number were random factors, and lead ratio was the predicted measure (Fig. 6).

Analysis of the model showed significant interaction between the fixed factors (*F*_(1, 576)_ = 5.6, *P* = .018, *η*_p_^2^ = .001. Further analysis of this model showed that in the spontaneous condition, the parents’ lead ratio (EMM = .143, SE = .009), was significantly greater (*t*_(576)_ = 4.86, *P* < .001) than the children’s (EMM = .083, SE = .009), whereas in the intentional condition no significant difference (*t*_(576)_ = 1.549, *P* = .122) between the children’s (EMM = .174, SE = .009) and the parents’ (EMM = .193, SE = .009) lead ratios was found. These findings suggest that parents tend to lead only in the spontaneous condition but not when the dyads are explicitly instructed to synchronize.

To examine whether age was associated with task performance and synchrony measures, we conducted Pearson correlation analyses between mean child age and each behavioral parameter, with *P*-values Bonferroni-corrected for multiple comparisons. Age was not significantly associated with any of the behavioral measures examined, including mean Euclidean distance (*r* = .007, *P* = 1.00), number of goals collected (*r* = .227, *P* = 1.00), obstacle collisions (*r* = −.209, *P* = 1.00), overall game score (*r* = .317, *P* = .504), perfect synchrony (*r* = .405, *P* = .117), lagged synchrony (*r* = −.208, *P* = 1.00), parent lead ratio (*r* = −.029, *P* = 1.00), or child lead ratio (*r* = −.157, *P* = 1.00).

### Neuroimaging analysis

To test our third hypothesis and determine whether interbrain synchrony reflected genuine dyadic coupling rather than random pairing, we compared 33 real dyads and 33 pseudo dyads using LME models. Mean WTC values (without baseline correction) served as the dependent variable. Fixed effects included condition, ROI, and dyad type (real vs. pseudo), with block number entered as a random effect. Three models were estimated: a main-effects model; a model including all two-way interactions; and a model including all interaction terms. Model comparisons using Type II Wald χ² tests indicated that the model including two-way interactions did not improve fit relative to the main-effects model, χ²_(97)_ = 83.31, *P* = .83, nor relative to the fully interactive model, χ²_(48)_ = 38.15, *P* = .84. Accordingly, subsequent analyses were based on the main-effects model.

Examination of the model revealed a significant main effect of dyad type (*F*_(1,62)_ = 19.03, *P* < .001), such that mean WTC values were significantly lower in the pseudo dyads (EMM = .222, SE = .02) compared with the real dyads (EMM = .341, SE = .02).

Having established stronger interbrain synchrony in real than pseudo dyads, we next examined whether synchrony relative to baseline was evident at specific ROI combinations and differed across task conditions. First, to explore the neural underpinnings of synchrony, we examined whether inter-brain synchrony (ΔWTC) differed from zero at different ROI combinations in both conditions. The LME model we used included condition (Spontaneous, Intentional) and ROI combination (49 in total) as fixed factors; ΔWTC as predicted value; and dyads’ ID numbers as a random factor; the predicted variables included children’s leadership values, and parents’ leadership values, taken from across the game (a separate model was constructed for each behavioral factor).

No significant interaction between ROI combination and condition was found (*F*_(48, 12448)_ = .533, *P* = .99, *η*_p_^2^ = .002). Similarly, no main effect of condition was found (*F*_(1, 12448)_ = .917, *P* = .338, *η*_p_^2^ < .001). A main effect of ROI combination was found (*F*_(48, 12448)_ = 2.435, *P* < .001, *η*_p_^2^ < .001). To examine which ROI combinations yielded synchrony above baseline we tested whether ΔWTC values in each ROI was higher than zero within the model, applying Bonferroni adjustment for multiple comparisons. We found that ΔWTC was significantly higher than zero in the following ROI combinations: rDLPFC-lDLPFC (EMM = .05, SE = .009, *P* < .001), rDLPFC_lIFG (EMM = .04, SE = .009, *P* < .001), rDLPFC_lParietal (EMM = .04, SE = .01, *P* < .001), rDLPFC_mPFC (EMM = .03, SE = .01, *P* = .043), rDLPFC_rDLPFC (EMM = .04, SE = .009, *P* < .001), rDLPFC_rIFG (EMM = .03, SE = .009, *P* < .001), rDLPFC_rParietal (EMM = .04, SE = .009, *P* < .001), lDLPFC_lDLPFC (EMM = .04, SE = .009, *P* < .001), lDLPFC_lParietal (EMM = .03, SE = .001, *P* = .02), lDLPFC_rDLPFC (EMM = .04, SE = .009, *P* < .001), lIFG_lParietal (EMM = .03, SE = .001, *P* = .013), lIFG_rDLPFC (EMM = .04, SE = .009, *P* < .001), lIFG_rIFG (EMM = .04, SE = .009, *P* < .001), lParietal_rDLPFC (EMM = .04, SE = .009, *P* < .001), mPFC_rIFG (EMM = .03, SE = .001, *P* = .024), rIFG_rDLPFC (EMM = .04, SE = .009, *P* = .002). The remaining 33 ROI combinations yielded no significantly higher than baseline synchrony during the task.

### Brain and behavior relationship

**Figure 7:**
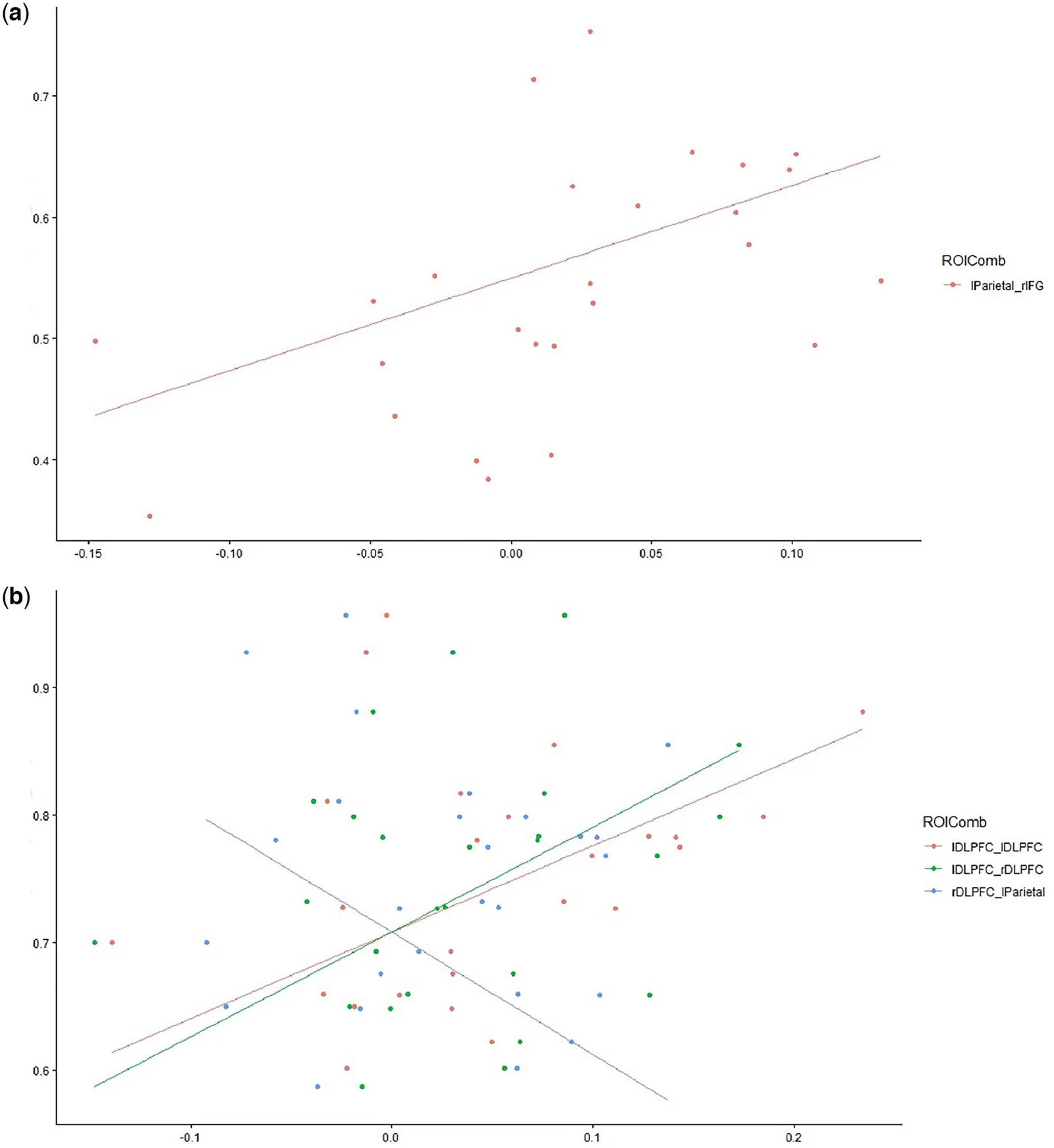
Sample scatterplots and regression lines of the ROI combinations in which ΔWTC was a significant predictor of behavioral synchrony in the final stage of the stepwise regression, for the spontaneous (a) and intentional (b) conditions.

As indicated above, 16 ROI combinations showed significant difference from baseline. To assess which of these 16 ROI combinations may predict behavioral synchrony (all sync measure), we conducted a multiple regression analysis. Two regression analyses were carried out and included the CVV all-sync measure as the predicted variable, and the ΔWTC values of the 16 ROI combinations in either the intentional or the spontaneous condition (Fig. 7). The regression model for the intentional condition was found to be significant, *F*_(5, 25)_ = 2.79, *P* = .046, Adj *R*^2^ = 0.263. Interbrain synchrony in three combinations was found to be significant predictor of behavioral synchrony. The significant predictors are as follows: lDLPFC-lDLPFC (*β* = .572, *t* = 2.72, *P* = .013), lDLPFC-rDLPFC (*β* = .599, *t* = 2.38, *P* = .026), rDLPFC-lParietal (*β* = −.635, *t* = −2.41, *P* = .026). The regression model for the spontaneous condition was found to be significant, *F*(3, 25) = 5.58, *P* = .005, Adj *R*^2^ = .355. Interbrain synchrony in one combination was found to be significant predictor of behavioral synchrony: lParietal_rIFG (*β* = .508, *t* = 3.04, *P* = .006).

Parallel analyses were conducted on HHb signals using the identical analytic pipeline applied to O_2_Hb. These results did not reveal significant brain–behavior associations and are reported in full in the Supplementary Material.

## Discussion

The present study examined how parent–child dyads synchronize behavior and neural activity across spontaneous and intentional conditions, with particular attention to leadership. In contrast to simplified motor tasks such as tapping or mirroring, the Flocking Game provided a child-friendly, dynamic paradigm embedding cooperation under competing demands, offering a naturalistic proxy for parent–child interaction.

Results indicated that even without explicit instruction, dyads showed significant spontaneous synchrony, indicating that parent–child pairs readily align during shared interaction. These findings align with evidence that synchrony is a fundamental, implicit process in social interaction and emerges in early parent–infant exchanges (Feldman 2007, Marton-Alper *et al.* 2020). When synchrony was explicitly instructed, it increased significantly, suggesting that intentional engagement amplifies dyadic alignment, consistent with evidence that task structure enhances interpersonal synchrony (Schmidt and Richardson 2008, Marton-Alper *et al.* 2023).

Beyond the general presence of synchrony, results revealed clear leadership asymmetries across conditions. In the spontaneous condition, parents showed a greater tendency to lead than their children, consistent with the hierarchical structure of the parent–child relationship, in which parents serve as role models and secure bases for exploration (Bowlby 1969, Popper and Mayseless 2003). When synchrony was explicitly instructed, this difference diminished, suggesting that structured contexts can equalize dyadic roles by reducing reliance on parental regulation. This pattern accords with attachment-based models proposing that leadership is context-sensitive and emerges most strongly under ambiguity or challenge, when children turn to trusted figures for guidance (Morgeson 2005). Similar effects have been observed in competitive versus cooperative parent–child play, where parents assume leadership under pressure or novelty, highlighting the regulatory function of parental guidance (Yarmolovsky and Geva 2024). Together, these findings suggest that parental leadership reflects a supportive regulatory role rather than a drive for control.

Contrary to our third hypothesis, we did not find greater overall interbrain synchrony in the intentional than in the spontaneous condition. Rather, synchrony above baseline emerged across both conditions, with no overall condition difference. This suggests that the distinction between conditions was not reflected in a global increase in neural coupling, but in more specific patterns of neural organization. To clarify these differences, we next examined which ROI combinations showed reliable synchrony and how they related to behavioral coordination.

We next identified parent–child ROI combinations showing interbrain synchrony significantly above baseline in both conditions. Sixteen region pairs exceeded baseline, indicating widespread neural coupling during parent–child interaction. A substantial proportion of these pairs involved the parent’s right and left DLPFC coupled with multiple child regions, which may indicate a prominent involvement of this region in supporting intentional synchrony. This pattern aligns with the DLPFC’s established role in goal-directed control, monitoring, and executive regulation during social interaction (Avnor *et al.* 2025, Sobeh and Shamay-Tsoory 2025).

In addition to DLPFC effects, consistent coupling was observed in the IFG and IPL, regions commonly associated with action observation processes (Su *et al.* 2020). These regions have been implicated in shared action mapping, mutual prediction, and perspective-taking in prior hyperscanning research (Numssen *et al.* 2021, Zhang *et al.* 2021), suggesting their contribution to the neural foundations of behavioral synchrony, including in spontaneous contexts.

Critically, of the sixteen region pairs, only three ROI combinations predicted behavioral synchrony in the intentional condition. Interbrain synchrony involving the parent’s left DLPFC and the child’s regions consistently showed positive associations with behavioral synchrony. The predominance of interbrain synchrony involving the parent’s DLPFC accords with the behavioral finding that parents tended to lead more than children. Leadership in this context involves monitoring and structuring the dyadic exchange, functions attributed to proactive regulatory mechanisms of the left DLPFC (Schmidt *et al.* 2018, White *et al.* 2023). Consistent with our hypothesis, intentional synchrony and leadership were associated with greater engagement of top-down monitoring within the parent’s fronto-parietal control system (Avnor and Shamay-Tsoory 2025).

The right DLPFC, in contrast, showed a negative predictive relationship. Whereas the left DLPFC has been linked to proactive goal maintenance and sustained top-down regulation (Braver *et al.* 2009), the right DLPFC has been associated with reactive and corrective control under conflict or uncertainty (Toghi *et al.* 2024). Reduced synchrony involving the right DLPFC therefore predicted better behavioral coordination, potentially reflecting a shift from reactive to smoother, proactive interaction.

In contrast to the intentional condition, in which DLPFC interbrain synchrony was dominant, the spontaneous condition showed that parent left IPL-child right IFG coupling predicted higher behavioral synchrony, consistent with the automatic nature of spontaneous alignment. The IPL and IFG are often described as core components of the action observation network and have been proposed to support mapping observed actions onto one’s own motor representations and shared understanding during unstructured interaction (Lestou *et al.* 2008, Stillesjö *et al.* 2025). This pattern may reflect spontaneous alignment processes through which synchrony emerges without explicit intention, relying on sensorimotor resonance and perspective alignment (Bhat *et al.* 2017).

Together, these findings point to a functional dissociation in how the two suggested neural systems relate to behavioral synchrony across contexts. During spontaneous synchrony, coupling within regions commonly associated with the action observation network, encompassing the IFG and IPL, was selectively related to behavioral synchrony, reflecting suggested bottom-up resonance mechanisms that may enable partners to naturally align their movements and internal states through mirroring and shared representations (Morales *et al.* 2019, Su *et al.* 2020). However, we acknowledge that activity in these regions does not necessarily reflect engagement of the entire action observation network, and alternative, non-social interpretations cannot be ruled out. Such automatic mapping may explain how synchrony can emerge organically without explicit instruction, consistent with prior evidence linking these regions to embodied imitation and perspective-taking (Basso *et al.* 2020, Korisky *et al.* 2020). Importantly, this interpretation represents only one possible functional account of the observed interbrain synchrony. Alternatively, coupling within the IFG, IPL, and lateral prefrontal regions may reflect coordinated attentional allocation and monitoring, given their overlap with dorsal and ventral attention systems supporting goal-directed and stimulus-driven attention (Corbetta and Shulman 2002). From this perspective, synchrony may index shared attentional engagement or reorienting during continuous visuomotor tracking of one’s own and the partner’s avatar, rather than mirroring or embodiment (Vossel *et al.* 2014).

In contrast, intentional synchrony engaged the fronto-parietal network, with the parents’ DLPFC, particularly the left hemisphere, serving as a candidate neural anchor for deliberate, goal-oriented synchrony. This pattern suggests that spontaneous and intentional synchrony are supported by partially distinct neural pathways, highlighting how parent–child coordination can emerge through different mechanisms depending on task demands.

### Limitations

Several limitations warrant consideration. First, the modest sample size may have limited statistical power to detect subtler or more complex effects; larger cohorts may reveal additional brain–behavior relationships. Second, the design was correlational, assessing associations between neural and behavioral synchrony; therefore, causal inferences cannot be drawn.

Third, the experimental design did not allow counterbalancing of condition order, as the spontaneous synchrony condition was necessarily administered before the intentional condition to prevent carryover effects from explicit coordination instructions. Although theoretically motivated, this sequencing may have influenced behavioral and neural measures; future studies could address this limitation through between-subject designs or alternative protocols to better control potential order effects.

Finally, although the gaming paradigm provided a controlled, ecologically inspired framework for eliciting spontaneous and intentional synchrony, it still approximates real-life interaction. Naturalistic parent–child exchanges likely involve richer contextual, emotional, and communicative components.

**Table 1:** ROI abbreviations and full anatomical names.

rIFG	Right inferior frontal gyrus
lIFG	Left inferior frontal gyrus
rIPL	Right inferior parietal lobule
lIPL	Left inferior parietal lobule
rDLPFC	Right dorsolateral prefrontal cortex
lDLPFC	Left dorsolateral prefrontal cortex
mPFC	Medial prefrontal cortex

## Supplementary Material

nsag066_Supplementary_Data

## Data Availability

Non-identifiable data and all analysis code supporting the findings of this study will be made publicly available upon publication via Mendeley Data (DOI: 10.17632/hsydph9wxz.1). All personal identifying information of the participants will not be published.

## References

[nsag066-B1] Ackerman JM, Bargh JA. 14 two to tango: Automatic social coordination and the role of felt effort. *Effortless Attention* 2010; 335.

[nsag066-B3] Avnor Y , Atias D, Markus A et al It takes two to empathize: interbrain coupling contributes to distress regulation. Emotion 2025;25:736–54. 10.1037/emo0001431 39531679

[nsag066-B4] Avnor Y , Shamay-Tsoory S. Abnormal interbrain coupling in individuals with childhood adversity may underlie their difficulties in benefiting from social interactions. J Affect Disord 2025;377:206–16. 10.1016/j.jad.2025.02.050 39986578

[nsag066-B5] Baayen RH , Davidson DJ, Bates DM. Mixed-effects modeling with crossed random effects for subjects and items. J Mem Lang 2008;59:390–412.

[nsag066-B6] Barbey AK , Colom R, Grafman J. Dorsolateral prefrontal contributions to human intelligence. Neuropsychologia 2013;51:1361–9. 10.1016/j.neuropsychologia.2012.05.017 PMC3478435 22634247

[nsag066-B7] Basso JC , Satyal MK, Rugh R. Dance on the brain: enhancing intra- and inter-brain synchrony. Front Hum Neurosci 2020;14:584312. 10.3389/fnhum.2020.584312 PMC7832346 33505255

[nsag066-B8] Bates D, Mächler M, Bolker B et al Fitting linear mixed-effects models using lme4. J Stat Softw 2015*;*67*:*1–48.

[nsag066-B9] Benjamini Y , Hochberg Y. Controlling the false discovery rate: a practical and powerful approach to multiple testing. J R Stat Soc Series B (Methodol) 1995;57:289–300.

[nsag066-B10] Bergen D. The role of pretend play in children’s cognitive development. Early Childhood Res Pract 2002;4:n1.

[nsag066-B11] Bernieri FJ, Rosenthal R. Interpersonal coordination: Behavior matching and interactional synchrony. In: Feldman RS, Rimé B (eds.) *Fundamentals of Nonverbal Behavior*. Cambridge University Press, 1991, pp. 401-432.

[nsag066-B12] Bhat AN , Hoffman MD, Trost SL et al Cortical activation during action observation, action execution, and interpersonal synchrony in adults: a functional near-infrared spectroscopy (fNIRS) study. Front Hum Neurosci 2017;11:431. 10.3389/fnhum.2017.00431 PMC5591977 28928646

[nsag066-B13] Biringen Z. Emotional availability: conceptualization conceptualization and research findings. Am J Orthopsychiatry 2000;70:104–14. 10.1037/h0087711 10702855

[nsag066-B14] Black B , Logan A. Links between communication patterns in mother‐child, father‐child, and child‐peer interactions and children’s social status. Child Dev 1995;66:255–71.

[nsag066-B15] Bosmans G , Van Vlierberghe L, Bakermans-Kranenburg MJ et al A learning theory approach to attachment theory: exploring clinical applications. Clin Child Fam Psychol Rev 2022;25:591–612. 10.1007/s10567-021-00377-x PMC8801239 35098428

[nsag066-B16] Bowlby J. (1969). Attachment and loss, Vol. 1: attachment. In Attachment and Loss. New York: Basic Books.

[nsag066-B17] Bowlby, J. The making and breaking of affectional bonds: I. Aetiology and psychopathology in the light of attachment theory. Br J Psych 1977;130:201–10. 10.1192/bjp.130.3.201 843768

[nsag066-B18] Braver TS , Paxton JL, Locke HS et al Flexible neural mechanisms of cognitive control within human prefrontal cortex. Proc Natl Acad Sci USA 2009;106:7351–6. 10.1073/pnas.0808187106 PMC2678630 19380750

[nsag066-B19] Brigadoi S , Cooper RJ. How short is short? Optimum source-detector distance for short-separation channels in functional near-infrared spectroscopy. Neurophotonics 2015;2:025005 10.1117/1.NPh.2.2.025005 PMC4478880 26158009

[nsag066-B20] Brumariu LE. Parent-child attachment and emotion regulation. New Dir Child Adolesc Dev 2015;2015:31–45. 10.1002/cad.20098 26086126

[nsag066-B21] Buccino G , Binkofski F, Riggio L. The mirror neuron system and action recognition. Brain Lang 2004;89:370–6. 10.1016/S0093-934X(03)00356-0 15068920

[nsag066-B22] Burns JM. Leadership. New York, NY: Open Road Media, 2012.

[nsag066-B23] Chartrand TL , Lakin JL. The antecedents and consequences of human behavioral mimicry. Annu Rev Psychol 2013;64:285–308. 10.1146/annurev-psych-113011-143754 23020640

[nsag066-B24] Corbetta M , Shulman GL. Control of goal-directed and stimulus-driven attention in the brain. Nat Rev Neurosci 2002;3:201–15. 10.1038/nrn755 11994752

[nsag066-B25] Cui X , Bray S, Reiss AL. Functional near infrared spectroscopy (NIRS) signal improvement based on negative correlation between oxygenated and deoxygenated hemoglobin dynamics. Neuroimage 2010;49:3039–46. 10.1016/j.neuroimage.2009.11.050 PMC2818571 19945536

[nsag066-B5731359] Faul F, , Erdfelder E, , Lang A-G et al G*power 3: a flexible statistical power analysis program for the social, behavioral, and biomedical sciences. Behav Res Methods 2007;39:175–91. 10.3758/bf03193146 17695343

[nsag066-B30] Feldman R. Parent-infant synchrony and the construction of shared timing; physiological precursors, developmental outcomes, and risk conditions. J Child Psychol Psychiatry 2007;48:329–54. 10.1111/j.1469-7610.2006.01701.x 17355401

[nsag066-B31] Fishburn FA , Ludlum RS, Vaidya CJ et al Temporal derivative distribution repair (TDDR): a motion correction method for fNIRS. Neuroimage 2019;184:171–9. 10.1016/j.neuroimage.2018.09.025 PMC6230489 30217544

[nsag066-B33] Gatti R , Rocca MA, Fumagalli S et al The effect of action observation/execution on mirror neuron system recruitment: an fMRI study in healthy individuals. Brain Imaging Behav 2017;11:565–76. 10.1007/s11682-016-9536-3 27011016

[nsag066-B34] Goldin PR , McRae K, Ramel W et al The neural bases of emotion regulation: reappraisal and suppression of negative emotion. Biol Psychiatry 2008;63:577–86. 10.1016/j.biopsych.2007.05.031 PMC2483789 17888411

[nsag066-B35] Grinsted A , Moore JC, Jevrejeva S. Application of the cross wavelet transform and wavelet coherence to geophysical time series. Nonlinear Process Geophys 2004;11:561566.

[nsag066-B36] Hardwick RM , Caspers S, Eickhoff SB et al Neural correlates of action: comparing meta-analyses of imagery, observation, and execution. Neurosci Biobehav Rev 2018;94:31–44. 10.1016/j.neubiorev.2018.08.003 30098990

[nsag066-B37] Harrist AW , Waugh RM. Dyadic synchrony: its structure and function in children’s development. Dev Rev 2002;22:555–92.

[nsag066-B38] Hove MJ , Risen JL. It’s all in the timing: interpersonal synchrony increases affiliation. Soc Cogn 2009;27:949–60.

[nsag066-B40] Iacoboni M , Woods RP, Brass M et al Cortical mechanisms of human imitation. Science 1999;286:2526–8. 10.1126/science.286.5449.2526 10617472

[nsag066-B41] Jiang J , Dai B, Peng D et al Neural synchronization during face-to-face communication. J Neurosci 2012;32:16064–9. 10.1523/JNEUROSCI.2926-12.2012 PMC6621612 23136442

[nsag066-B42] Jones JD , Cassidy J, Shaver PR. Parents’ self-reported attachment styles: a review of links with parenting behaviors, emotions, and cognitions. Pers Soc Psychol Rev 2015;19:44–76. 10.1177/1088868314541858 PMC4281491 25024278

[nsag066-B43] Korisky A , Eisenberger NI, Nevat M et al A dual-brain approach for understanding the neuralmechanisms that underlie the comforting effects of social touch. Cortex 2020;127:333–46. 10.1016/j.cortex.2020.01.028 32259669

[nsag066-B46] Lestou V , Pollick FE, Kourtzi Z. Neural substrates for action understanding at different description levels in the human brain. J Cogn Neurosci 2008;20:324–41. 10.1162/jocn.2008.20021 18275338

[nsag066-B47] Marton-Alper IZ , Gvirts-Provolovski HZ, Nevat M et al Herding in human groups is related to high autistic traits. Sci Rep 2020;10:17957. 10.1038/s41598-020-74951-8 PMC7578000 33087785

[nsag066-B48] Marton-Alper IZ , Markus A, Nevat M et al Differential contribution of between and within‐brain coupling to movement synchronization. Hum Brain Mapp 2023;44:4136–51. 10.1002/hbm.26335 PMC10258530 37195028

[nsag066-B49] Mayseless O , Popper M. Attachment and leadership: review and new insights. Curr Opin Psychol 2019;25:157–61. 10.1016/j.copsyc.2018.08.003 30107317

[nsag066-B50] Miles LK , Griffiths JL, Richardson MJ et al Too late to coordinate: contextual influences on behavioral synchrony. Euro J Social Psych 2010;40:52–60.

[nsag066-B53] Morales S , Bowman LC, Velnoskey KR et al An fMRI study of action observation and action execution in childhood. Dev Cogn Neurosci 2019;37:100655. 10.1016/j.dcn.2019.100655 PMC6570413 31102960

[nsag066-B54] Morgeson FP. The external leadership of self-managing teams: intervening in the context of novel and disruptive events. J Appl Psychol 2005;90:497–508. 10.1037/0021-9010.90.3.497 15910145

[nsag066-B55] Numssen O , Bzdok D, Hartwigsen G. Functional specialization within the inferior parietal lobes across cognitive domains. Elife 2021;10:e63591. 10.7554/eLife.63591 PMC7946436 33650486

[nsag066-B57] Palumbo RV , Marraccini ME, Weyandt LL et al Interpersonal autonomic physiology: a systematic review of the literature. Pers Soc Psychol Rev 2017;21:99–141. 10.1177/1088868316628405 26921410

[nsag066-B59] Pinti P , Tachtsidis I, Hamilton A et al The present and future use of functional near-infrared spectroscopy (fNIRS) for cognitive neuroscience. Ann N Y Acad Sci 2020;1464:5–29. 10.1111/nyas.13948 PMC6367070 30085354

[nsag066-B60] Pollonini L , Bortfeld H, Oghalai JS. PHOEBE: a method for real time mapping of optodes-scalp coupling in functional near-infrared spectroscopy. Biomed Opt Express 2016;7:5104–19. 10.1364/BOE.7.005104 PMC5175555 28018728

[nsag066-B61] Popper M , Mayseless O. Back to basics: applying a parenting perspective to transformational leadership. Leadersh Q 2003;14:41–65.

[nsag066-B62] Reiss PT , Gvirts HZ, Bennet R et al A time-varying measure of dyadic synchrony for three-dimensional motion. Multivariate Behav Res 2019;54:530–41. 10.1080/00273171.2018.1547874 30957565

[nsag066-B63] Repp BH , Penel A. Rhythmic movement is attracted more strongly to auditory than to visual rhythms. Psychol Res 2004;68:252–70. 10.1007/s00426-003-0143-8 12955504

[nsag066-B64] Rizzolatti G , Craighero L. The mirror-neuron system. Annu Rev Neurosci 2004;27:169–92. 10.1146/annurev.neuro.27.070203.144230 15217330

[nsag066-B67] Schmidt RC , Bienvenu M, Fitzpatrick PA et al A comparison of intra-and interpersonal interlimb coordination: coordination breakdowns and coupling strength. J Exp Psychol Hum Percept Perform 1998;24:884–900. 10.1037//0096-1523.24.3.884 9627423

[nsag066-B68] Schmidt L , Tusche A, Manoharan N et al Neuroanatomy of the vmPFC and dlPFC predicts individual differences in cognitive regulation during dietary self-control across regulation strategies. J Neurosci 2018;38:5799–806. 10.1523/JNEUROSCI.3402-17.2018 PMC6246877 29866743

[nsag066-B69] Schmidt RC , Richardson MJ. Dynamics of interpersonal coordination. In Coordination: Neural, Behavioral and Social Dynamics. Berlin, Heidelberg: Springer, 2008, 281–308.

[nsag066-B71] Sobeh A , Shamay-Tsoory S. The emergence of moral alignment within human groups is facilitated by interbrain synchrony. Commun Biol 2025;8:464. 10.1038/s42003-025-07831-4 PMC11926081 40114031

[nsag066-B72] Stillesjö S , Hjärtström H, Johansson AM et al Action execution and observation in autistic adults: a systematic review of fMRI studies. Autism Res 2025;18:238–60. 10.1002/aur.3291 PMC11826028 39673256

[nsag066-B74] Su WC , Culotta ML, Hoffman MD et al Developmental differences in cortical activation during action observation, action execution and interpersonal synchrony: an fNIRS study. Front Hum Neurosci 2020;14:57. 10.3389/fnhum.2020.00057 PMC7062643 32194385

[nsag066-B75] Su WC , Culotta M, Mueller J et al Differences in cortical activation patterns during action observation, action execution, and interpersonal synchrony between children with or without autism spectrum disorder (ASD): an fNIRS pilot study. PLoS One 2020;15:e0240301. 10.1371/journal.pone.0240301 PMC7595285 33119704

[nsag066-B76] Tarr B , Launay J, Dunbar RI. Silent disco: dancing in synchrony leads to elevated pain thresholds and social closeness. Evol Hum Behav 2016;37:343–9. 10.1016/j.evolhumbehav.2016.02.004 PMC4985033 27540276

[nsag066-B77] Toghi A , Chizari M, Khosrowabadi R. A causal role of the right dorsolateral prefrontal cortex in random exploration. Sci Rep 2024;14:24796. 10.1038/s41598-024-76025-5 PMC11493979 39433838

[nsag066-B78] Toga AW , Thompson PM. Mapping brain asymmetry. Nat Rev Neurosci 2003;4:37–48. 10.1038/nrn1009 12511860

[nsag066-B82] Vossel S , Geng JJ, Fink GR. Dorsal and ventral attention systems: distinct neural circuits but collaborative roles. Neuroscientist 2014;20:150–9. 10.1177/1073858413494269 PMC4107817 23835449

[nsag066-B83] Wager TD , Smith EE. Neuroimaging studies of working memory. Cogn Affect Behav Neurosci 2003;3:255–74. 10.3758/cabn.3.4.255 15040547

[nsag066-B84] White LK , Makhoul W, Teferi M et al The role of dlPFC laterality in the expression and regulation of anxiety. Neuropharmacology 2023;224:109355. 10.1016/j.neuropharm.2022.109355 PMC9790039 36442650

[nsag066-B85] Yarmolovsky J , Geva R. Follow the leader: parent-and child-led synchrony in competitive and cooperative play. J Nonverbal Behav 2024;48:235–51.

[nsag066-B86] Zhang M , Jia H, Wang G. Interbrain synchrony of team collaborative decision-making: an fNIRS hyperscanning study. Front Hum Neurosci 2021;15:702959. 10.3389/fnhum.2021.702959 PMC8319628 34335212

[nsag066-B87] Zimeo Morais GA , Balardin JB, Sato JR. fNIRS optodes’ location decider (fOLD): a toolbox for probe arrangement guided by brain regions-of-interest. Sci Rep 2018;8:3341. 10.1038/s41598-018-21716-z PMC5820343 29463928

