# Supplementary material for: Parental leadership in motion: neural and behavioral mechanisms of synchrony during parent–child interaction
Source: Soc Cogn Affect Neurosci. 2026 Aug 8;21(1):nsag066. doi: 10.1093/scan/nsag066 (PMC13509953; doi:10.1093/scan/nsag066)
Supplement: Supplementary file 1 [file nsag066_supplementary_data.docx]

**Supplemental Material:**

**HHb Brain-Behavior Relationship**

In addition to the analyses conducted on oxygenated hemoglobin (O₂Hb), we performed parallel analyses on deoxygenated hemoglobin (HHb) signals in order to examine whether similar brain-behavior relationships would emerge across hemoglobin measures. Using the same analytic pipeline applied to the O₂Hb data, we first examined which interbrain ROI combinations showed task-related HHb synchrony significantly greater than zero.

To examine which ROI combinations yielded synchrony above baseline, we tested whether ΔWTC values in each ROI combination were higher than zero within the model, applying Bonferroni adjustment for multiple comparisons. This analysis revealed that ΔWTC was significantly higher than zero in the following eight interbrain ROI combinations: rIFG-rDLPFC (EMM = .03, SE = .008, p = .004), lIFG-mPFC (EMM = .03, SE = .009, p = .007), rDLPFC-lDLPFC (EMM = .03, SE = .008, p = .007), mPFC-lParietal (EMM = .03, SE = .009, p = .007), lIFG-rIFG (EMM = .03, SE = .008, p = .008), mPFC-rDLPFC (EMM = .03, SE = .009, p = .016), rParietal-lIFG (EMM = .03, SE = .008, p = .038), and mPFC-rParietal (EMM = .03, SE = .009, p = .041). The remaining ROI combinations did not show HHb synchrony significantly greater than baseline during the task.

These eight ROI combinations were subsequently entered as predictors in stepwise multiple regression analyses, with behavioral synchrony (CVV All Sync) serving as the dependent variable. As in the O₂Hb analyses, separate regression models were constructed for the spontaneous and intentional synchrony conditions.

For the spontaneous synchrony condition, the regression model including HHb-based interbrain synchrony predictors did not reach statistical significance, F_(2, 23)_ = 3.11, p = .064. Thus, none of the HHb ROI combinations significantly predicted behavioral synchrony in the intentional condition.

Similarly, for the intentional synchrony condition, the regression model was not significant, F_(1, 24)_ = 2.86, p = .104, indicating that no HHb-based interbrain synchrony measures reliably predicted behavioral synchrony in this condition either.

Given that none of the HHb regression models reached significance, no individual HHb ROI combinations were interpreted or reported as predictors of behavioral synchrony. Accordingly, our interpretations were focused on the O₂Hb findings, which showed a condition-specific brain-behavior associations.
